# Impact of atrial fibrillation on outcomes of patients treated by transcatheter mitral valve repair: A systematic review and meta-analysis

**DOI:** 10.1097/MD.0000000000022195

**Published:** 2020-10-02

**Authors:** Fuqiang Sun, Honghao Liu, Qi Zhang, Fanfan Lu, Haibo Zhan, Jiawei Zhou

**Affiliations:** a Department of Cardiovascular Surgery; b Department of Emergency; c Department of Endovascular Surgery, the First Affiliated Hospital of Zhengzhou University, Zhengzhou, China.

**Keywords:** atrial fibrillation, prognosis, transcatheter mitral valve repair

## Abstract

**Background:**

Conflicting data have been reported related to the impact of atrial fibrillation (AF) on outcomes after transcatheter mitral valve repair with MitraClip (MC) implantation. In this study, we assessed the prognosis of MC-treated patients according to the presence of pre-existing AF.

**Methods:**

Randomized and observational studies reporting outcomes of pre-existing AF or sinus rhythm in patients undergoing MC treatment were identified with an electronic search. Outcomes of interest were short-and long-term mortality, stroke, bleeding, rehospitalization, myocardial infarction (MI), cardiogenic shock, acute procedure success, the hospital stay, and the number of Clips implanted.

**Results:**

Eight studies (8466 individuals) were eligible. Compared to sinus rhythm, long-term mortality, the risk of bleeding, rehospitalization, and longer hospital stay were significantly higher in AF groups, whereas similar correlations were found in the analysis of other outcomes.

**Conclusion:**

AF may be related with worse outcomes in patients undergoing MC implantation, including long-term mortality, major bleeding, and rehospitalization. AF should be taken into account when referring a patient for MC treatment.

## 1 Introduction

Mitral regurgitation (MR) is the most common valvular heart disease, with over 3 million people in the United States having moderate-to-severe or severe MR.^[1]^ Severe MR confers an adverse prognosis, with a survival comparable to or even worse than that of most advanced malignancies.[^2^,^3^] Approximately half of patients with severe MR are not treated because of advanced age, reduced left ventricular function, co-morbidities, or other contraindications for open mitral valve surgery.^[4]^ Although mitral valve surgery is curative for primary MR, neither surgical repair nor replacement of the mitral valve has been shown to lower the rate of hospitalization or death associated with secondary mitral regurgitation, and both procedures confer a substantial risk of complications.[^5^,^6^]

Transcatheter mitral valve repair with the MitraClip system is now an established treatment option in patients with severe MR, and the results from different clinical trials in recent years have proved that MC implantation is effective in alleviating heart failure symptoms and improving the health status of patients with severe MR who remain symptomatic despite maximally tolerated guideline-directed medical therapy.[^7^,^8^] One previous independent registry shows that MC is associated with high immediate success, low complication rates, and sustained 1-year reduction of severity of mitral regurgitation of clinical symptoms.^[9]^ AF is the most common arrhythmia and is frequently present in patients referred for surgery for MR.^[10]^ AF is associated with adverse cardiovascular events and decreased survival, and is a strong predictor of death during follow-up, even after adjusting for other known risk factors and for class I recommendations for MV surgery.^[11]^ In recent trials and registries, AF was found in 31.7% to 67.7% of patients undergoing MC implantation.[^12^,^13^] However, the impact of AF on the clinical outcomes of patients with MR undergoing MC implantation has not been determined.

Interestingly, there are conflicting data regarding the effect of preoperative AF on the outcome of MC implantation,[^14^,^15^,^16^] and currently, the impact of AF on the results of patients with TAVI treatment have been confirmed using the method of meta-analysis,^[17]^ no systematic review or meta-analysis exists to address this question in MC implantation, therefore, the objective of this study was to assess the role of preoperative AF in patients undergoing MC implantation using a meta-analysis approach.

## 2 Methods

### 2.1 Literature search

Since the study is a secondary literature study based on the published studies, no ethical approval is required.

We performed a systematic review and meta-analysis according to recommendations of the Preferred Reporting Items for Systematic Reviews and Meta-Analyses statement.^[18]^ Systematic searches using Embase and PubMed databases were carried out to identify potential studies, with keywords including “mitraclip,” “mitral clip,” “percutaneous mitral valve repair,” “edge-to-edge,” “Alfieri,” and “atrial fibrillation” until November 2019. The reference lists of included studies were further assessed for the supplement.

### 2.2 Study selection and data extraction

The obtained citations were initially evaluated by two independent researcher at the title and/or abstract level, and the final consensus was critical for the divergences. When potentially pertinent, the following explicit selection criteria and study inclusion criteria were used as follows:

(a) Published in peer-reviewed journals;
(b) All patients were treated percutaneously using the MC;
(c) Reported comparative information between patients with and without AF before MC implantation;
(d) Study should provide the details of hazard ratio (HR) or odds ratio (OR), or adequate data for calculation of these measures.

The full texts of the selected studies were subsequently reviewed by the same investigators independently. The following articles were excluded, such as case reports, reviews, meta-analyses, animal studies, overlapped or duplicated publications, and studies or conference abstracts without sufficient data for the calculation of measurements and 95% CI.

A pre-piloted form was used during the extraction of data by the 2 reviewers independently (HHL and QZ). Original data of demographic elements and endpoints were obtained from the main text and supplementary materials in the studies. Missing data were requested through email to the corresponding author, and only manuscripts providing the latest information were included in cases of studies with overlapping populations. Any discrepancies between the reviewers were resolved through consensus or consultation with a third reviewer (FQS).

### 2.3 Outcomes of interest and definition

Our primary outcomes were in-hospital and long-term mortality. Moreover, we considered short-term occurrence of stroke (hemorrhagic and ischemic), postoperative major bleeding, myocardial infarction and cardiogenic shock. In addition, we also considered the long-term occurrence rate of rehospitalization. In-hospital mortality and long-term mortality were defined as all-cause mortality within 30 days after the operation and 12 months later. Stroke was defined as the occurrence of a new stroke confirmed by CT. In patients with preoperative stroke, postoperative stroke was defines as worsening of the neurologic deficit with new radiologic findings. Major bleeding was defined as bleeding that need re-exploration or blood product transfusions. Cardiogenic shock was defined as inadequate blood flow due to the dysfunction of the ventricular of heart.

### 2.4 Methodological assessment

We used the Newcastle–Ottawa Scale (NOS) to conduct the methodological evaluation for the observational properties of all included studies. Studies graded with more than 6 scores were identified as high-quality trials, and meanwhile, a revised Jada Scale was employed to evaluate randomized trials. Studies rated with four marks or more were recognized as high-quality in methodology. Each study was appraised by two evaluators independently. Any disagreement was resolved by negotiated settlement.

### 2.5 Statistical analysis

MD and Hedge G estimators were used for meta-analysis of continuous data. Survival was assessed by hazard ratio (HR) and its 95% confidential interval (95% CI), and when Kaplan–Meier curves were provided instead of HR, two researchers independently estimated the HR indirectly from the curve using Engauger Digitizer version 9.0 according to the methods described by Tierney et al.^[19]^ Risk ratios (RR) were calculated for dichotomous data, representing the odds that an outcome will occur given a particular exposure compared with the odds of the outcome occurring in the absence of that exposure. All analyses were conducted using Stata 14.0 (College Station, TX). A fixed-effects inverse variance model was used throughout. Heterogeneity was quantified using the Cochran Q-statistic and I^2^ index tests, and 25% was considered to indicate significant heterogeneity. Sensitivity analysis was conducted by removing low-quality studies and interchanging calculation models (fixed-effects and random-effects) in order to observe outcome stability. Publication bias was assessed using visual inspection of funnel plots, and by Egger and Begg regression, and was considered significant if found to be present in all tests. Statistical significance was indicated by *P* < .05.

## 3 Results

### 3.1 Baseline characteristics

**Figure 1:**
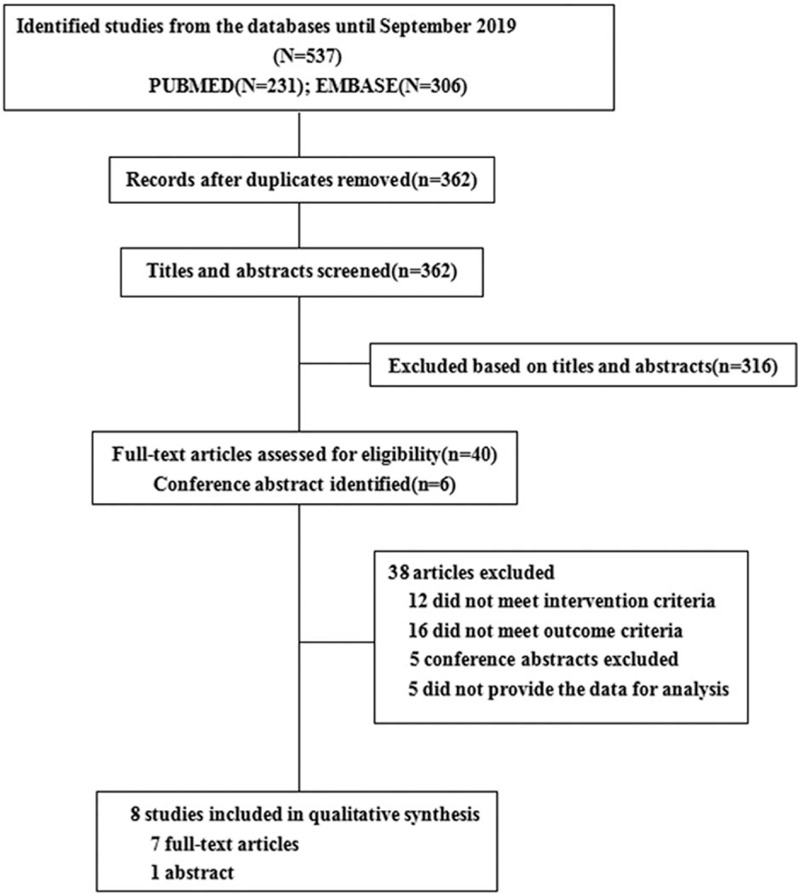
Flow chart of meta-analysis.

A total of 537 records were identified with the initial search, after deduplication, 362 citations remained, and then, 316 publications were ignored after screening the title and abstract. 6 congress abstracts and 40 full-texts of the remaining 46 references were obtained and assessed according to our predetermined inclusion and exclusion criteria. Ultimately, seven published articles[^14^,^15^,^16^,^20^,^21^,^22^,^23^] and one study^[24]^ presented at the EUROECHO 2016 conference published between 2012 and 2019 were included in our analysis. Figure 1 shows the flow chart of the study selection process.

The principle characteristics of the included studies are summarized in Table 1, and Table 2 shows the baseline characteristics of included patients. Of the eight included articles, one was randomized, two were retrospective, and the remaining five were prospective analyses. All the studies were based on western populations; three were conducted in both America and Germany, and one in both Italy and France. The duration of follow-up ranged between 1 and 5 years. A total of 8466 patients undergoing the MC procedure were analyzed, 4994 patients with AF and 3472 without AF (NAF). According to Table 2, Patients with AF were significantly older than those without AF (years, 79.1 ± 6.9 vs 74.4 ± 4.3, *P* < .001), and other perioperative characteristics were comparable between groups.

**Table 1:**
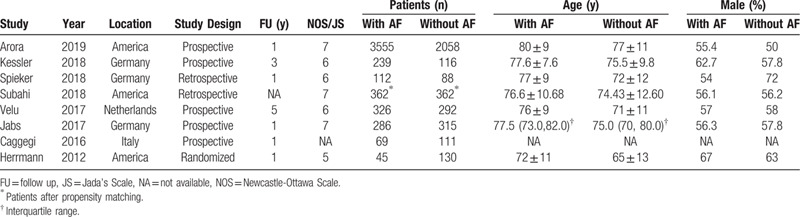
Selected studies for review and meta-analysis.

**Table 2:**
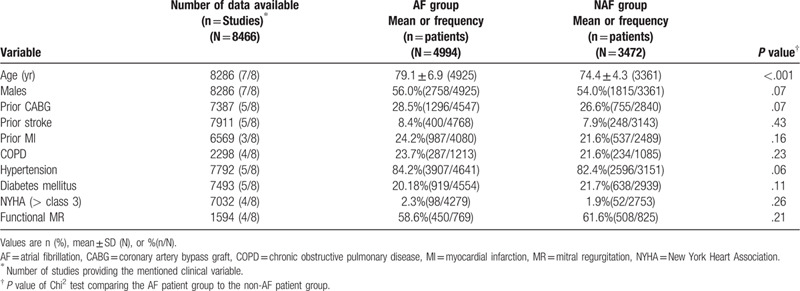
Baseline clinical profile of included patients.

### 3.2 Mortality

**Figure 2:**
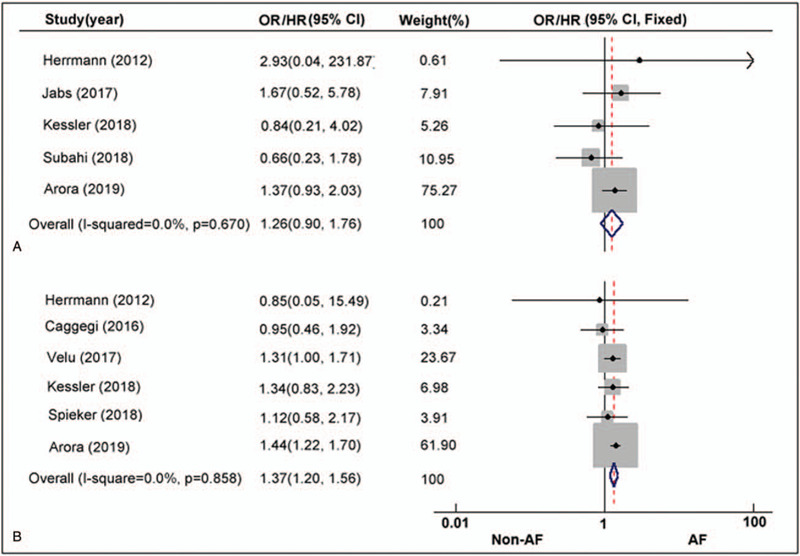
AF vs Non-AF. Risk ratio with 95% CI for the composite endpoint of in-hospital mortality; (B) Hazard ratio with 95% CI for the composite endpoint of long-term survival.

Five of the 8 included studies reported the incidence of in-hospital mortality. In total, 121 of 4487 (2.7%) patients in the AF group had died in hospital compared with 64 of 2981 (2.1%) of the NAF patients. The OR for the comparison was 1.26 (95% CI: 0.90–1.76, *P* = .18; I^2^ = 0.0%, *P* = .67; Fig. 2A), indicating that there was no statistically significant difference in in-hospital all-cause mortality between the 2 groups. The data of long-term survival were derived from studies with a follow-up of over one-year. As shown in Figure 2B, 6 studies provided survival information directly or indirectly. The merged outcome indicated that patients with AF had a statistically significant 1.37-fold HR increase in all-cause death (HR: 1.37; 95% CI: 1.20–1.56, *P* < .01) with an absence of heterogeneity across the studies (I^2^ = 0.0%, *P* = .86).

### 3.3 Stroke

**Figure 3:**
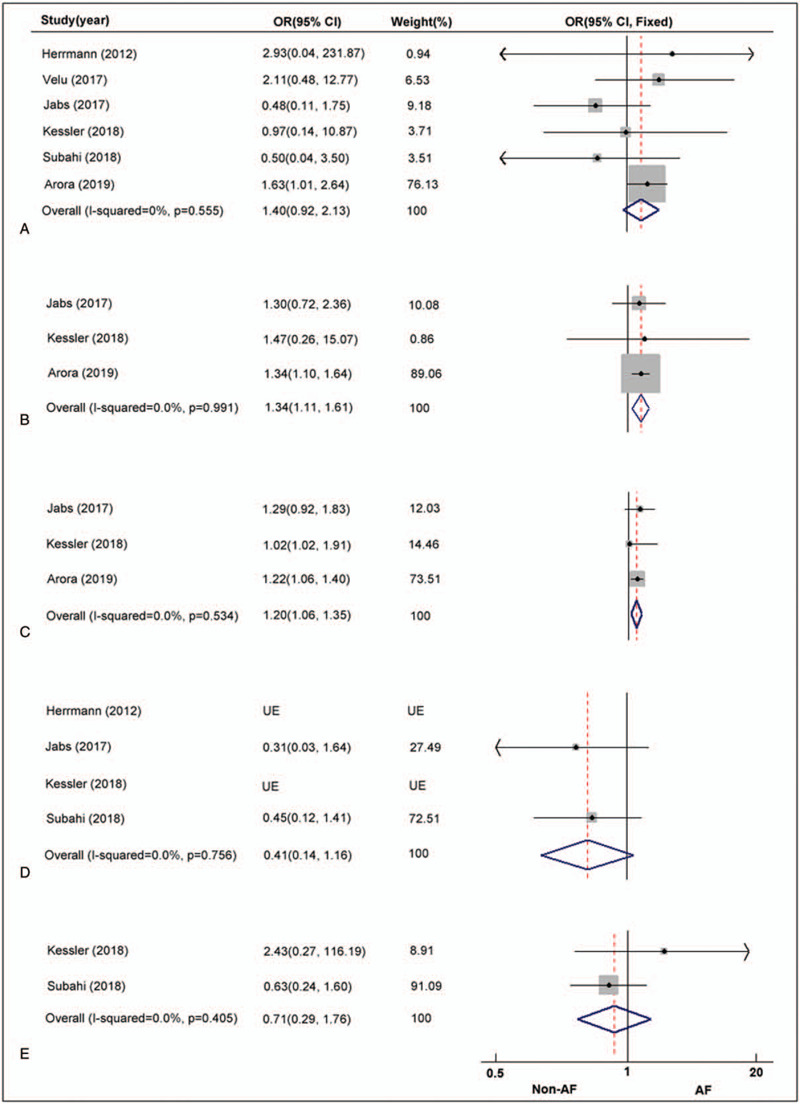
AF vs NAF. Risk ratio and 95% CI for the pooled endpoints of stroke (A), bleeding (B), rehospitalization (C), myocardial infarction (D) and cardiogenic shock(E).

Six studies reported the in-hospital stroke rate. The pooled result suggested that there was no significant difference in the odds of stroke occurring in the AF group compared to the NAF group; although a trend toward higher rates of stroke was seen in patients with preoperative AF, it failed to reach statistical significance (OR: 1.40, 95% CI: 0.92–2.13, *P* = .11; I^2^ = 0.0%, *P* = .56; Fig. 3A).

### 3.4 Bleeding

Three articles reported the postoperative in-hospital major bleeding incidence. As shown in Figure 3B, we identified a higher rate of bleeding in the AF group with an OR of 1.34, meaning that in patients with AF, there was a significant 34% odds ratio increase in postoperative bleeding events compared with the absence of AF (OR: 1.34, 95% CI: 1.11–1.61, *P* < .01; I^2^ = 0.0%, *P* = .99).

### 3.5 Rehospitalization

The rehospitalization rate was calculated for cases of heart failure or other life-threatening situations at 1-year follow-up. Data were available from 3 studies. In total, 1017 of 4080 (24.9%) patients in the AF group experienced rehospitalization events compared with 597 of 2489 (23.9%) NAF patients. We found that the presence of AF was associated with a 20% increase in rehospitalization rate (OR: 1.20, 95% CI: 1.06–1.35, *P* < .01; I^2^ = 0.0%, *P* = .53; Fig. 3C).

### 3.6 Myocardial infarction

The postoperative myocardial infarction (PMI) end point was reported by 4 out of the 8 included articles. However, we were unable to calculate ORs due to insufficient information provided in 2 studies.[^16^,^20^] PMI was reported only in 25 out of 1325 patients (1.9%), without any difference between patients with AF and sinus rhythm (OR: 0.41, 95% CI: 0.14–1.16, *P* = 0.09; I^2^ = 0.0%, *P* = .76; Fig. 3D).

### 3.7 Cardiogenic shock

Only 2 studies reported the postoperative cardiogenic shock end point, with a total of 29 events found in 1078 patients. The pooled result failed to identify a significant difference between the 2 groups (OR: 0.71, 95% CI: 0.29–1.76, *P* = .46; I^2^ = 0.0%, *P* = .41; Fig. 3E).

### 3.8 Acute procedure success

**Figure 4:**
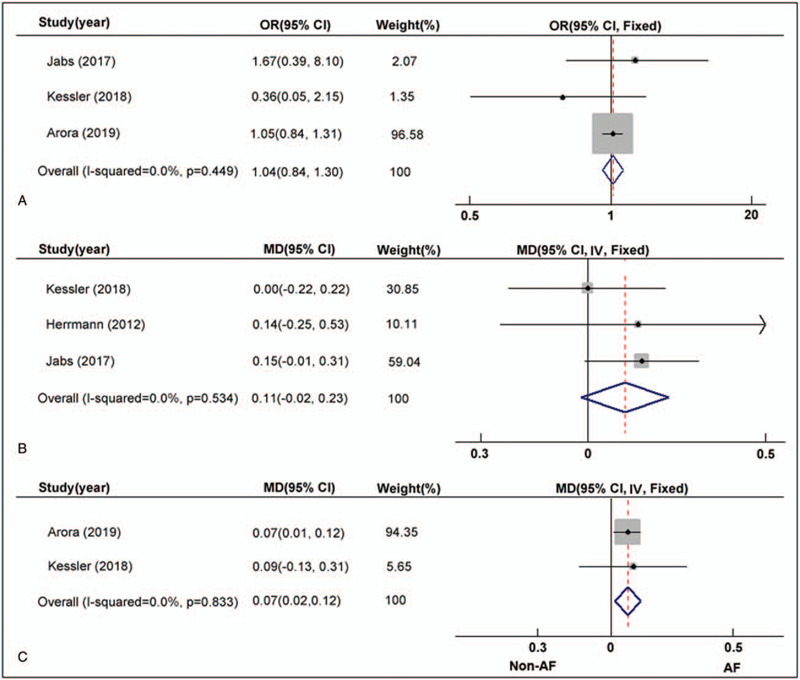
AF vs NAF. Risk ratio and 95% CI for the comparison of procedure failure. (B) MD and 95%CI for the pooled endpoint of number of clip implanted. (C) MD and 95% CI for the comparison of hospital stay.

The end point of acute procedure success was defined as non-severe mitral regurgitation (MR degrade < 2) after placement of the clips. In total, 254 out of 4080 (6.2%) patients had a failed procedure in the AF group compared with 144 of 2489 (5.8%) patients in the NAF group, and there was no significant difference in acute procedure success (OR: 1.04, 95% CI: 0.84–1.30, *P* = .69; I^2^ = 0.0%, *P* = .45; Fig. 4A).

### 3.9 Number of clips implanted

Three included studies reported the number of clips implanted. As shown in Figure 4B, the pooled result indicated that the number of clips implanted was similar in patients with AF and patients without AF (MD: 0.11, 95% CI: -0.02–0.23, *P* = .09; I^2^ = 0.0%, *P* = .53).

### 3.10 Hospital stay

As shown in Figure 4C, 2 studies reported the comparison of hospital stay end point. After meta-analysis, we found that AF was associated with a slightly longer length of hospital stay during the index hospitalization for transcatheter MV repair (MD: 0.07, 95% CI: 0.02–0.12, *P* = .01; I^2^ = 0.0%, *P* = .83).

### 3.11 Risk of bias and sensitivity analysis

**Figure 5:**
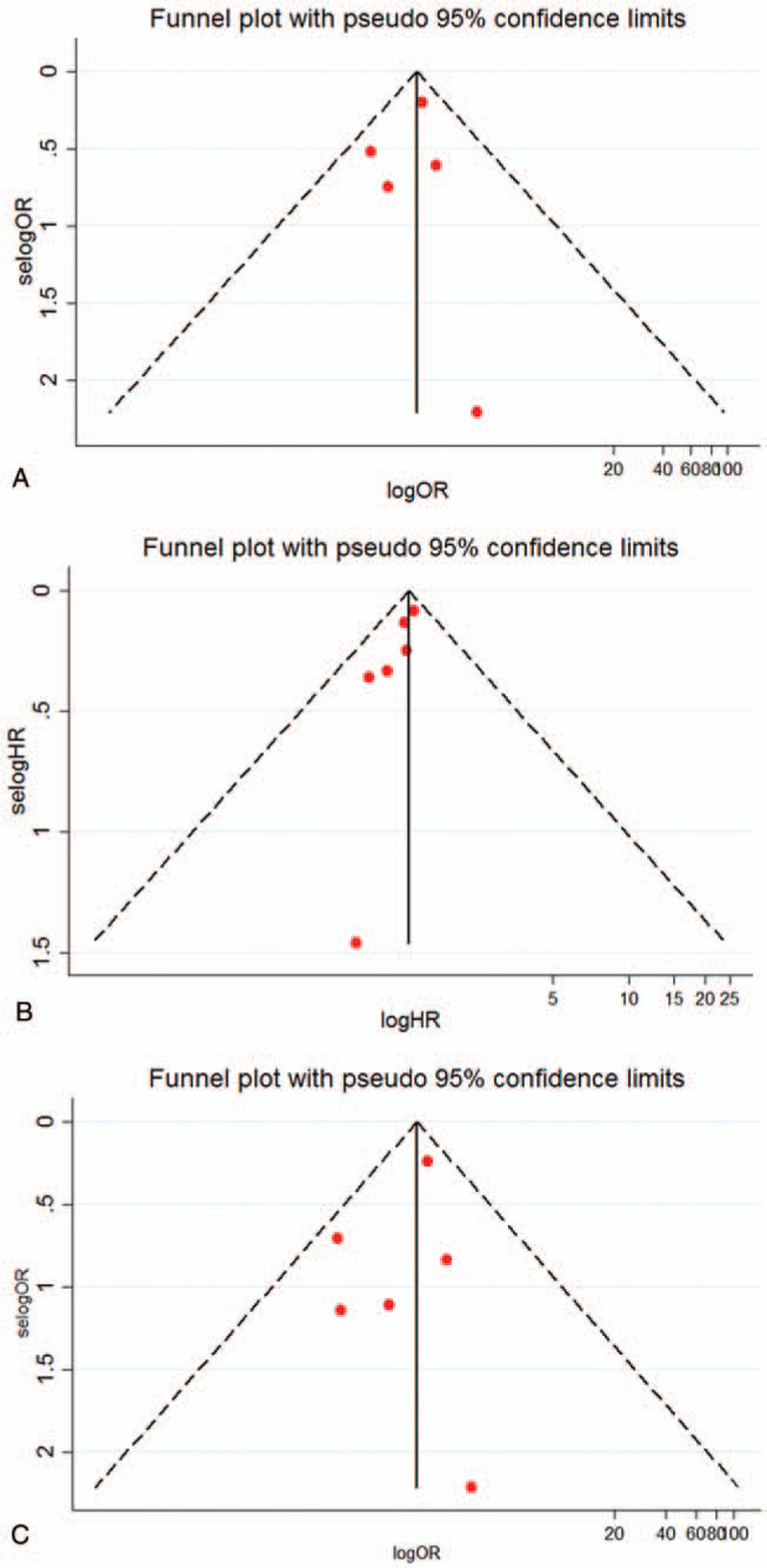
Funnel plot of the meta-analysis. AF vs NAF with endpoints of in-hospital mortality (A), long-term survival (B) and stroke (C).

**Figure 6:**
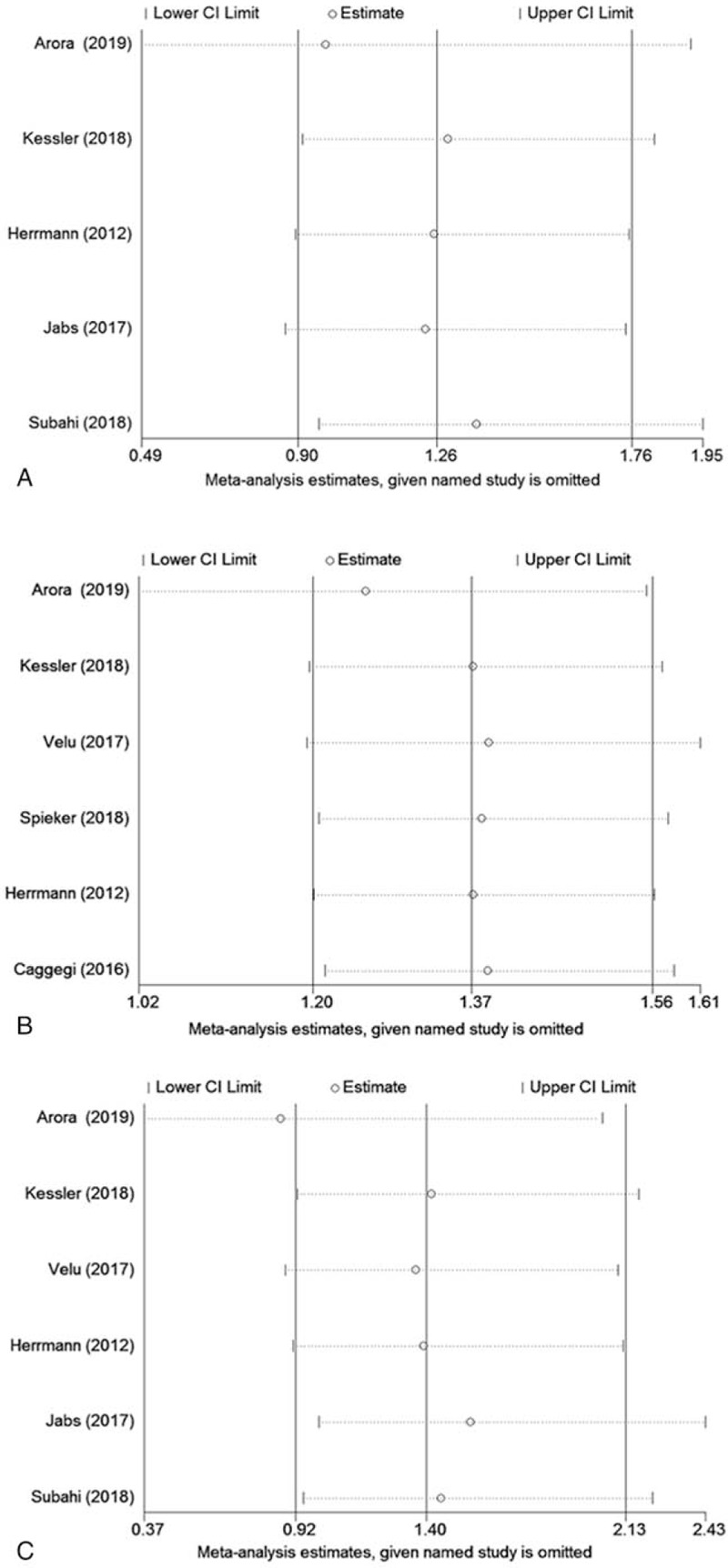
Sensitivity analysis for in-hospital mortality (A), long-term survival (B), and stroke (C).

As shown in Table 1, by the Newcastle–Ottawa Scale, all the prospective and retrospective studies were confirmed as high-quality trials in methodology (NOS > 6), and the only RCT was also appraised as being of high methodological quality (score of 5). We did not find any significant publication bias in the main outcomes of in-hospital death (Egger test: *P* = .695; Begg test: *P* = .806; Fig. 5A), long-term mortality (Egger test: *P* = .04; Begg test: *P* = .26; Fig. 5B) or stroke (Egger test: *P* = .39; Begg test: 0.71. Fig. 5C) when comparing patients with and without AF. We also performed a sensitivity analysis for the three main outcomes, and the association between AF and these endpoints were confirmed; none were had a significant influence on the results of this meta-analysis (Fig. 6A–C).

## 4 Discussion

To our knowledge, this is the updated and most comprehensive meta-analysis assessing the impact of AF on clinical outcomes after transcatheter mitral valve repair. One previous study completed by De Rosa et al found AF negatively affected LV reverse remodeling and 1-year survival after MC treatment in patients with both heart failure and functional mitral regurgitation.^[25]^ Although AF is an established risk factor for adverse cardiovascular events and decreased survival following surgical repair or mitral valve replacement,[^26^,^27^] actually, its impact on MR individuals undertaking MC implantation has not been well studied. With the remarkable increase in MC use, it is crucial to verify the effect of AF on clinical outcomes after MC implantation.

The main results of this study-level meta-analysis of a large population of 8466 patients were:

1: AF is correlated to an increased risk of all-cause mortality at long-term follow-up; however, the presence of AF at baseline does not seem to represent a risk factor for in-hospital all-cause mortality following MC;

2: post-procedure outcomes such as the incidence of stroke, myocardial infarction, cardiac shock, acute procedure success and the number of clips implanted were similar between AF and NAF patients;

3: AF is associated with increased risk of major bleeding, rehospitalization, and longer length of hospital stay.

Enlarged left atria seemingly lead to AF in patients with severe MR, while the remediation of sinus rhythm in patients with AF has been revealed to alleviated MR intensity.^[28]^ AF as MR is an advanced pathological progress, which might trigger or even magnify MR degree, while severe MR could also activate AF. Although present studies still can’t expound the details, it is now identified that MR and AF was linked by fundamental association, which is important to the assessment prior to MC implantation.^[29]^ Unfortunately, only the Endovascular Valve Edge-to-Edge Repair Study II (EVEREST II) RCT, which included 264 total patients, has been designed to characterize patients with MR and AF treated percutaneously using the MC device, and pre-existing AF was present in just 27% of patients.^[16]^ Data on the impact of AF on outcomes after MC remain scarce and conflicting. To fully understand the role of AF in MC implantation, we examined the significant differences in this meta-analysis, which pooled the results of all studies reporting comparisons between patients with and without AF.

In our study, in-hospital mortality was similar in both subgroups with no statistical difference detected. Among the 5 studies included in our pooled analysis, despite the higher risk profile characterizing patients with AF that predisposed them to worse outcomes, none of them showed a significantly increased in-hospital mortality rate in AF patients.

The low incidence of death for both groups underscores the safety of MC in “real-world” practice regardless of the initial rhythm. The rate of in-hospital death was 2.7% and 2.1% for patients with and without AF, respectively, in our analysis, which is consistent with that reported in the TVT Registry (2.3% overall for patients)^[30]^ but slightly lower than the 3.4% in the ACCESS-EU study; this discrepancy can be explained by the baseline characteristics of the ACCESS-EU cohort, which had a higher risk profile compared with most populations enrolled in other registries, with a higher mean age, more patients with NYHA Class III or IV, and higher burden of comorbidities.^[31]^ It is important to note that AF increased the risk of all-cause mortality by a statistically significant 37% during the long-term follow-up (over one year) in AF patients who received MC. Almost no studies showed no significantly increased mortality rates in AF patients compared to NAF patients. Nevertheless, after merging, the pooled HR showed that the difference was statistically significant, though it is difficult to determine whether the presence of AF had a negative impact on survival or if it was just a marker of sicker patients. The AF group was associated with more advanced comorbidities, including older age and lower LVEF, which are known to be risk factors of poorer outcomes after MC implantation.^[23]^ Therefore, to what extent AF is involved in the progress of postoperative adverse events, as well as the role of confounding factors such as aged and antithrombotic treatment remains to be clarified. In the present analysis, patients in AF groups were older than those in NAF groups, however, we were not able to consider the effects of age on outcomes because the data provided in studies were not sufficient to complete a subset analysis, and thus, age may be a confounding factor that ultimately influences our results in this study. As such, the extent of decreased survival due to AF noted from observational studies needs to be taken with caution. A previous study has demonstrated that, compared to the sinus rhythm group, patients with preoperative AF have significantly reduced survival, with AF being an independent negative predictor of survival in multivariate analysis.^[32]^ A recent meta-analysis of 23 studies, including 3253 functional MR patients undergoing MC, found an adverse impact of AF on 1-year survival; however, the correlation was not confirmed at 2 years. Conversely, the EVEREST II trail found no difference in all-cause mortality at 12 months between patients with and without AF.^[16]^ In the present meta-analysis, studies were not selected based on the ability to isolate MR etiology. FMR and DMR are two conditions that differ from each other in several ways, but one recent meta-analysis found that 1-year overall mortality was not significantly different between groups (FMR vs DMR, RR 1.26; 95% CI: 0.90 to 1.77; *P* = .18);^[33]^ accordingly, decreased survival due to AF should not be confounded by MR etiology in our study. Actually, once AF develops, it most likely bring about an enhancing degree of fibrosis (i.e., AF begets AF), which subsequently leads to an increased substrate for AF, furthermore, this vicious cycle probably results in continual deterioration of the AF burden regardless of surgical treatment and decreased survival compared to patients without AF.^[34]^

The association of AF with a higher risk for stroke is well known,^[35]^ however, our findings showed that the stroke rate was similar between patients with and without AF. The pooled OR indicated a trend of increased risk of stroke in patients with AF, but the difference was not statistically significant. This finding is important for concerns regarding thrombus formation after MC, and recent registry data suggest that the use of Non-vitamin K oral anticoagulant (NOAC) with a single antiplatelet drug may prove beneficial as compared with antiplatelet therapy alone,^[36]^ but this has not been confirmed by any clinical trials. When interpreting this discrepancy, the heterogeneity of antithrombotic regimens reported in studies should be considered. In addition, new-onset AF (NOAF) may be another causal factor, since a previous study including 2580 patients undergoing MC demonstrated that 57.2% of them developed NOAF, which seems to be associated with an increased risk of peripheral vascular disease.^[37]^ However, our study lacked information on anticoagulant agents and NOAF, and therefore the precise reasons for the previous results were not apparent.

In our study, patients with AF had a higher risk for bleeding compared with those without AF. This may be partially explained by the much more frequent need for antithrombotic and/or anticoagulant therapy in AF patients.^[23]^ One study demonstrated a bleeding event in approximately 20% of patients, with the majority of events involving a nonvascular access bleed.^[38]^ Therefore, some caution should be utilized prior to placing these high-risk patients back on full anticoagulation, especially for those with AF after MC implantation. We found that AF status at baseline was associated with a higher rate of rehospitalization, consistent with the study by Korber et al.^[38]^ This should not be a surprise since AF seems to be a surrogate for worse clinical status and more advanced HF stage. One recent study showed that rehospitalization correlated significantly with all-cause mortality and cardiovascular mortality;^[39]^ therefore, effective home care and maximized pharmacological treatment for HF are critical, particularly with AF patients undergoing MC implantation. A slightly longer length of hospital stay was identified in AF patients, perhaps also the result of baseline characteristics of AF patients. Other outcomes of cardiogenic shock, MI, and acute procedure success were similar in patients with or without AF in our analysis, but the interpretation of these results needs to be exercised with caution due to the limited number of included studies. Furthermore, in spite of the fact that the removal of the largest study^[23]^ did not significantly affect the overall summary estimate in the sensitivity analysis, the meta-analysis still might have been dominated by the single study (Arora et al^[23]^), which accounted for an approximately 70% weight in the analysis. Therefore, further carefully conducted randomized clinical trials are needed to examine the potential impact of AF on patients with MC implantation.

## 5 Study limitations

The present article represents a study-level meta-analysis; therefore, one relevant limitation is the lack of patient-level data. Data from 7 observational studies (including one conference abstract) and only one RCT were used, and the potential for residual confounding factors due to the observational nature of this research cannot be excluded. The association of AF and increased mortality is multifactorial in patients undergoing MC implantation,^[40]^ and hereof, AF might appear either as a determining element of adverse events or a just as a marker for more advanced heart disease. Nevertheless, more well-designed studies are desired to elucidate the determinants of this association. The data reported were not sufficient to analyze the role of AF subtypes and NOAF, so it is therefore unclear what effect, if any, the AF subtype or NOAF may have on the outcome of MC. This study was also limited by failing to distinguish the etiology of MR and the severity grading, which may bias the estimated results. As a result, more data, preferably in the form of randomized, large-scale, and carefully conducted trails, are necessary to explore the impact of AF on the clinical outcomes of patients undergoing MC.

## 6 Conclusion

In the present meta-analysis with more than 8000 patients included undergoing MC implantation, AF might be related with postoperative worse events, including long-term mortality, bleeding, rehospitalization, and hospital stay, whereas the rates of in-hospital death, stroke, MI, cardiogenic shock, acute procedure success and number of implanted clips may be comparable with those of patients without AF. This study suggests that AF should be carefully considered in the selection of patients as candidates for MC and during follow-up, and we hope that our results will assist such decision-making. Moreover, further studies are needed to clarify whether AF is merely a marker of increased risk or an independent risk factor in patients treated by MC implantation.

## Acknowledgments

The authors thank the National Natural Science Foundation of China for supporting our study.

## Author contributions

**Conceptualization**: Fuqiang Sun

**Data curation**: Fuqiang Sun, Honghao Liu, Qi Zhang.

**Formal analysis**: Fanfan Lu, Haibo Zhan, Jiawei Zhou.

**Writing**: Fuqiang Sun.
